# Employment and the youth mental health crisis in Canada: distinct influences across phases of the school-to-work transition

**DOI:** 10.3389/fpubh.2025.1601463

**Published:** 2025-07-31

**Authors:** Véronique Dupéré, Mathieu Caron-Diotte, Nancy Beauregard, Clémentine Courdi, Jiseul Sophia Ahn, Elizabeth Olivier, Kristel Tardif-Grenier, David Litalien

**Affiliations:** ^1^École de psychoéducation, Université de Montréal, Montréal, QC, Canada; ^2^Centre de recherche en santé publique, Montréal, QC, Canada; ^3^Institut universitaire Jeunes en difficultés, Montréal, QC, Canada; ^4^École de relations industrielles, Université de Montréal, Montréal, QC, Canada; ^5^Département de pédagogie et d’andragogie, Université de Montréal, Montréal, QC, Canada; ^6^Département de psychoéducation et psychologie, Université du Québec en Outaouais, Saint-Jérôme, QC, Canada; ^7^École de counseling et d’orientation et Département des fondements et pratiques en éducation, Université Laval, Québec, QC, Canada

**Keywords:** young adults, mental health, employment, school-to-work-transition, education

## Abstract

**Objectives:**

Employment-related challenges and uncertainties are thought to contribute to the mental health crisis affecting young adults globally. Yet, few studies have examined how employment characteristics relate to young adults’ mental health. This study addresses this gap, considering how the role of employment might vary depending on young adults’ educational status and level.

**Methods:**

A representative sample of 6,700 young adults (18–30 y.o.) drawn from Statistics Canada’s *Longitudinal and International Study of Adults* (2012–2020) was used to examine links between working hours and employment in a study-related job and mental health (life satisfaction, global mental health, psychological distress), beyond prior mental health and background characteristics. Interactions with student status were also incorporated.

**Results:**

Among young adults in tertiary (university, college) education, mental health worsened as working hours increased. For all other participants, the opposite was observed: working more hours was associated with improved mental health. Overall, the best outcomes were observed among tertiary-enrolled students not working, and the worst among youth neither working nor in education. The size of the differences between these groups were non-negligible (with *d* ranging between 0.37 and 0.47). Across all groups, employment in a study-related job was marginally associated with higher life satisfaction (but not with psychological distress or global mental health).

**Conclusion:**

Working hours contribute to young adults’ mental health in contrasting ways, depending on their position on the school-to-work transition continuum. Enhancing young adults’ access to meaningful employment in study-related jobs with an adapted schedule could help mitigate the youth mental health crisis.

## Introduction

Since the 2010s, young adults’ mental health has been on the decline globally, and this negative trend accelerated in the 2020s, in the wake of the COVID 19 pandemic (1, 2). This phenomenon has been documented in many countries, including in Canada, where the proportion of young adults aged 18 to 34 years reporting very good or excellent mental health dropped by 21 percentage points between 2015 and 2021, a sharp decline that left this age group below all others (3); see also Vaillancourt et al. (4). This situation has brought health authorities, globally and in Canada, to declare a state of crisis (1, 2, 5). According to a recently appointed Lancet Psychiatry Commission on the topic, employment supports should be an integral part of any plan targeting this crisis, considering the critical importance of career development in early adulthood (1); see also McGorry et al. (2) and Schweizer et al. (6). Youth advocacy groups concur and cite better employment support as a priority (7, 8). Recent guidelines for collaborative mental health care also highlight the need to develop services that address the social determinants of health, including access to meaningful employment, in collaboration with workplaces and educational institutions (9). Policy and service developments in that domain require a solid understanding of how employment, underemployment, or lack of employment affects young adults’ mental health.

Yet, little is known about how basic employment characteristics, such as working hours, relate to young adults’ mental health. A recent systematic review identified only four studies examining links between working conditions (e.g., working hours or contract type) and young workers’ mental health, among which only one was conducted in Canada (10, 11). Thus, using nationally representative longitudinal data, this study investigates the association between work hours and employment in a study-related job and young adults’ mental health, while considering their educational status, a key factor likely to modify the meaning and impact of employment.

### Working in early adulthood and mental health

Work relates to mental health in complex ways. When jobs are stable, decent, meaningful, and aligned with life goals, work is a mental health pillar; conversely, work erodes well-being when employment is lacking, precarious, unstable, unsafe, unsatisfactory, or encroaching on other social roles (12, 13). Although these patterns concern all workers, those under 30 years old are particularly affected, albeit in varying ways depending on where they are along the school-to-work transition continuum (14, 15).

#### Working after education

After exiting education, almost everyone wishes to rapidly find a full-time, decent job aligned with their educational credentials and life goals. Securing such a job is more challenging now than it was for previous generations, owing to profound labor market transformations (14). These challenges expose young adults who struggle to find decent, full-time employment to downstream consequences years later, including reduced career prospects and earnings (16, 17).

Besides earnings, employment-related challenges might also affect mental health. One indication of this is the recurrently high rate of mental health problems among youth not in education, employment, or training [NEET (13)]. While the mental health correlates of NEET situations are relatively well documented although more longitudinal research is needed; Gariépy et al. (13), much less is known about the impact of *under*employment, for instance, how young adults working part-time compare to those working full-time. In the one Canadian study on the topic cited in a recent review (11), young adults who transitioned into a full-time job after having completed their studies had fewer depression symptoms than those not working full-time, although the gap between the two groups narrowed over time (10).

#### Working while pursuing tertiary education

As knowledge-based sectors constantly expand, greater proportions of young adults look to improve their job prospects by staying in education longer. This is especially true in Canada, which has the highest proportion of college and university graduates among G7 nations (18). To finance these longer educational journeys, work increasingly becomes an important part of life *before* completing education. Recent estimates indicate that about 80% of students in Canada work during their postsecondary studies (19), a rate over 10% higher than a decade earlier (20).

Among young adults in university or college programs, the several hours of homework typically assigned for each hour spent in the classroom might be difficult to clock for those heavily involved in paid employment. Besides being inherently stressful, such time pressures can jeopardize young adults’ capacity to attain their educational goals and trigger feelings of failure and inadequacy. According to a Canadian representative longitudinal study, university students accumulating more than 24 h of paid work per week were less likely to complete their study program (20). These findings align with reviews and meta-analyses concluding that intensive work involvement can reduce chances of university program completion (21–23).

Far fewer studies have examined the mental health—as opposed to educational—consequences of working while studying, and their results are mixed. For instance, one large survey of 70,000 students enrolled in 120 U. S. postsecondary institutions found that working hours were associated with a sense of overload, but not with depression symptoms (24). The lack of longitudinal studies *directly* assessing links between working hours and mental health further obscures clear-cut conclusions. Relevant longitudinal studies infer effects *indirectly*, by focusing on students with financial concerns who tend to work more hours (25, 26). Students with such concerns tend to experience steeper mental health declines over time than unexposed peers, but it is unclear whether this drop reflects the weight of financial worries itself, or the consequences of actions taken to alleviate them, like working intensively.

Although working intensively can affect students’ educational and potentially mental health outcomes, studies also highlight that working while studying is not always problematic. When working hours are reasonable, working is often unrelated to negative educational outcomes, but rather to positive consequences, especially when jobs are aligned with students’ field of study, through processes of work-study enrichment (27).

#### Working while pursuing vocational, trades, or apprenticeship studies

Not all young adults in education are enrolled in tertiary programs where homework constitutes a major coursework component. A substantial proportion instead engage in vocational, trade, or apprenticeship programs where learning mostly occurs through internships and hands-on activities conducted during school hours. Among 25-to-34-year-olds in Canada, close to one in ten cite a trade or apprenticeship degree or certificate as their highest educational credential (28).

In such programs, paid work might not interfere with academics as much as in tertiary education. Although recent reviews cite no Canadian studies examining the impact of working hours among young adults in vocational, trade, or apprenticeship programs, results from other countries (e.g., Germany) suggest that working hours may not negatively affect educational outcomes among these students compared to peers in tertiary programs (21). If these trends also apply in Canada, then working hours, even at relatively intensive levels, might not undermine vocational students’ mental health, especially if the nature of the job is related to their field of study.

### The present study

This study aims to examine how working hours relate to young adults’ mental health in Canada, and whether this association varies for students at different levels of education compared to non-students. Based on previous research, it is expected that working hours will be associated with better mental health for young adults no longer in education, but with worse mental health among students. However, this negative association is expected to be strongest for students in tertiary education and lower or null among peers in vocational-type programs. Regardless of educational status, employment in a job related to one’s field of study should support mental health.

## Methods

### Sample

Data came from Statistics Canada’s *Longitudinal and International Study of Adults* (LISA), a representative panel survey of Canadians aged 15 and above living in the 10 provinces (29). In LISA, data collection waves occurred every two years from 2012 to 2020. As the variables of interest were only included from 2014 onward, the sample included respondents aged between 18 and 30 at the 2014, 2016, 2018 and 2020 waves. The resulting sample is approximately 6,700 unique respondents with 34,000 data points. Note that approximate sample sizes are given, as per Statistics Canada’s diffusion rules for products based on the LISA datasets.

### Measures

#### Mental health outcomes

The Kessler Psychological Distress Scale (30) measured *psychological distress* with 10 items asking about the frequency in the past month (0 = *Never* to 4 = *All the time*) of depression and anxiety symptoms (e.g., “Nervous,” “Hopeless,” “Sad or depressed”), with adequate internal consistency at each wave (*ω* ≥ 87). A higher score on this scale indicates greater psychological distress. Single items commonly used in Canadian surveys (31) measured *global mental health* (“In general, would you say your mental health is: …”; 0 = *Poor* to 5 = *Excellent*) and *life satisfaction* (“How do you feel about your life as a whole right now?”; 0 = *Very dissatisfied* to 10 = *Very satisfied*). These three measures were moderately correlated (| *r* | = 0.45 to 0.60).

#### Independent variables

Based on a series of questions regarding current educational enrollments, self-reported *educational enrolment status* at the time of the survey was coded as a three-level categorical variable [0 = Not in education (64%), 1 = In vocational education (trade schools, apprenticeships, technical or vocational education; 9%), 2 = In tertiary education [university or pre-university colleges, (27%)]. A continuous variable representing *working hours* was derived from a question asking respondents who declared having worked during the reference period their usual number of hours worked per week at all jobs (*M* = 32.0 h, *SD* = 17.5 h). A value of zero hours was attributed to those (9%) not working. For *employment in a study-related job*, a binary variable indicated whether individuals reported occupying a job related to their current or past field of studies [0 = No, 1 = Yes (46%)]. This last indicator was available only in the 2016 wave.

#### Sociodemographic and mental health controls

Participants reported their *age* [in years; *M* = 24.2, *SD* = 3.6], *sex* [0 = Male, 1 = Female (48%)], *visible minority status* [0 = Not belonging to a visible minority group, 1 = Visible minority (26%)]. *Family income* (divided into quintiles within each wave), and *immigrant generation status* [0 = Non-immigrant (62%), 1 = First-generation (14%), 2 = Second-generation (24%)] were obtained via data linkages between the LISA datasets and administrative records. *Previous mental health* was captured with the same measures as those described in the outcome section, but administered two years prior (i.e., in the preceding data collection wave).

### Analytic strategy

#### Full sample models focusing on working hours

Clustered models (with robust standard errors, conducted with the R software *sandwich* package) were used to account for the data’s nested structure [multiple data points per individual; Ntani et al. (32)]. Separate models regressed each outcome on the independent variables (weekly working hours, educational enrolment), their interaction, and controls, including previous mental health. Quadratic terms (with orthogonal polynomials) for work hours were also included to test for nonlinear associations (i.e., a positive effect of work hours until a threshold). As these quadratic terms were never significant, they were removed from the final models. Sex-stratified models were also conducted. As the results did not meaningfully differ between men and women, only non-stratified models are presented.

Population weights were applied to ensure representativity. Before fitting the models, data was initially screened for outliers using the median absolute deviation technique; univariate outliers on working hours (i.e., above 45 h) were replaced with a limit score. Missing data was handled with multiple imputation (*m* = 50) at the item level. Models were performed on all imputed datasets and their results were pooled using Rubin’s rule (33).

#### Subsample models incorporating involvement in a study-related job

A series of clustered models was also fitted to explore associations between mental health and involvement in a study-related job, using a reduced subsample of participants with valid data for the 2016 wave (the only one including the study-related job variable) and who had a job at this wave (*N* observations = 2,000). As power was reduced, especially for interactions involving low-frequency categories, a two-step approach was used. First, only the main effects (i.e., without interactions) of the independent variables (educational enrolment, working hours and involvement in a study-related job) were included, alongside controls [past mental health (2014), sociodemographics]. Second, three-way interactions between the independent variables were incorporated.

## Results

### Working hours (full sample models)

Results from the full sample models focusing on working hours are presented in Table 1. Results were replicated with mixed effect models but are not presented to avoid repetition, as they closely resembled those obtained in the retained models. Among participants not in education (reference category), working hours were associated with better mental health (lower levels of psychological distress, better global mental health, higher levels of life satisfaction).

**Table 1:** Coefficients for the clustered linear models predicting mental health.

	Distress		Global mental health		Life satisfaction	
	*b*	*SE*	*b*	*SE*	*b*	*SE*
Controls						
Age	−0.04	0.04	−0.01	0.01	0.01	0.01
Female (ref.: male)	1.20***	0.26	−0.22***	0.04	0.17**	0.06
Household income	−0.28***	0.08	0.03**	0.01	0.05*	0.02
Immigration generation (ref.: 3rd+)						
First generation	−1.59**	0.56	0.33***	0.08	0.23	0.14
Second generation	−0.39	0.40	0.06	0.05	0.09	0.07
Visible minority (yes vs. no)	−0.58	0.41	0.03	0.06	−0.30***	0.08
Previous mental health score	−1.28***	0.09	0.20***	0.01	0.42***	0.02
Main variables						
Education (ref: Not in education)						
Vocational education	−0.10	0.97	0.20	0.14	0.29	0.23
Tertiary education	−2.34***	0.60	0.36***	0.09	0.74***	0.13
Work hours (WH)	−0.04***	0.01	0.01***	0.00	0.01***	0.00
WH by educational enrolment						
WH x Vocational	0.00	0.03	0.00	0.00	−0.01	0.01
WH x Tertiary	0.08***	0.02	−0.01***	0.00	−0.02***	0.00
Pseudo *R*^2^	0.17***		0.17***		0.24***	

*p ≤ 0.05; **p < 0.01; ***p < 0.001.

**Figure 1:**
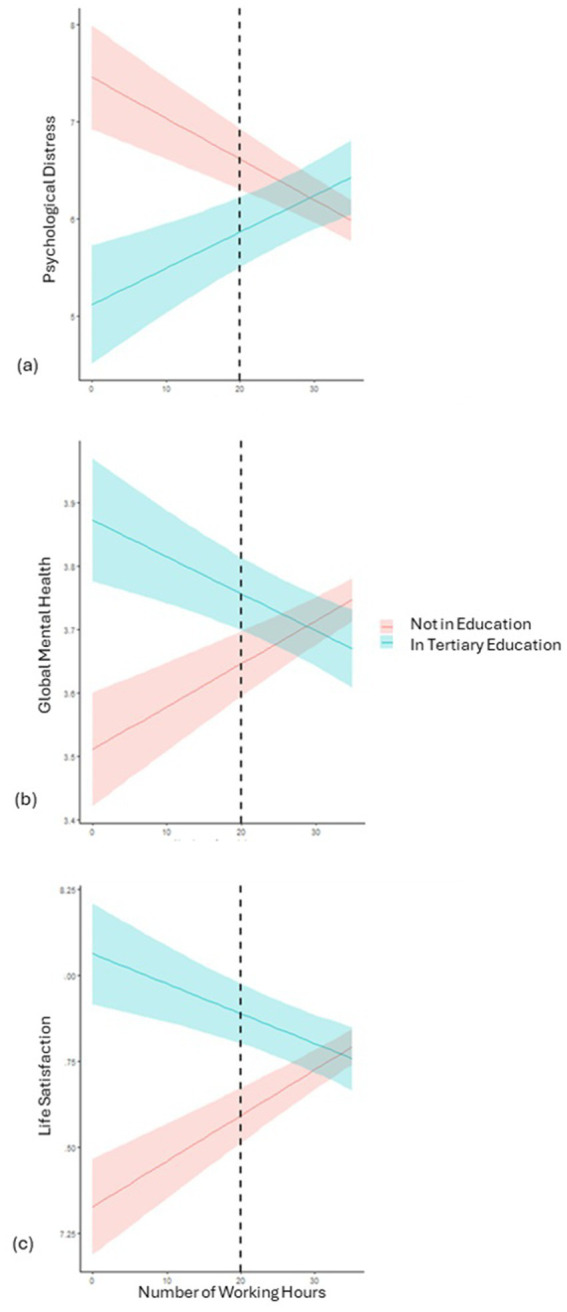
Association between the number of hours worked weekly and **(a)** psychological distress, **(b)** global mental health, and **(c)** life satisfaction, depending on educational enrolment (not in education, in tertiary education), while controlling for previous mental health score and holding other predictors constant.

Significant interactions were found for those in tertiary education, indicating that working hours were differentially associated with all mental health outcomes in this group compared to peers not in education, as shown in Figure 1. For these students, as working hours increased, mental health tended to deteriorate (increasing levels of psychological distress, diminishing global mental health and life satisfaction). When working at most moderately (i.e., below about 20 h per week, see Figure 1), they had significantly better mental health than peers not in education. These differences were widest when comparing non-working tertiary students to NEET youth (i.e., not in education and working 0 h), with effect sizes of *d* = 0.40 for psychological distress, *d* = 0.37 for global mental health, and *d* = 0.47 for life satisfaction. However, differences in mental health by educational enrolment status gradually diminished, and became non-significant for young adults’ workings longer hours (from 20 h and above, see Figure 1 and Supplementary Figure S1).

For those in vocational education, results yielded no significant main effects or interactions. That is, the association between work hours and mental health outcomes followed similar patterns to those of young adults not in education. The predicted mental health outcomes of those in vocational education generally fell between the two other groups (see Supplementary Figure S1).

### Employment in a study-related job (subsample models)

Step-1 main effects models showed that in the 2016 wave, having a study-related job was marginally associated with higher life satisfaction (*b* = 0.22, *SE* = 0.11, *p* = 0.051), even after controlling for past (in 2014) mental health, and the other independent and control variables. However, it was not associated with psychological distress (*b* = −0.37, *SE* = 0.43, *p* = 0.390), nor global mental health (*b* = 0.01, *SE* = 0.07, *p* = 0.890). In step-2 models incorporating three-way interaction terms, none of the interaction terms involving engagement in a study-related job were significant (results not shown). Thus, its marginal association with life satisfaction did not vary depending on educational enrolment or working hours.

## Discussion

In a representative sample of young Canadian adults, this study assessed associations between working hours and employment in a study-related job and mental health (i.e., psychological distress, global mental health, life satisfaction). These associations were investigated after adjusting for past mental health, and while considering educational enrolment (not in education; vocational education; tertiary education). The results showed that working hours were associated with young adults’ mental health in all subgroups, but in opposite directions depending on education enrolment status and level. Also, among students and non-students alike, employment in a job related to one’s current or past studies was marginally associated with higher life satisfaction (but not with other outcomes).

### Young adults in the process of establishing their careers warrant more attention from mental health scholars

Most young adults in the sample were no longer in education. Among this group, working full-time seemed protective for mental health. Indeed, those working more hours per week fared better than peers working fewer hours or not working at all. In fact, non-student youth who were unemployed or marginally employed systematically had the poorest mental health outcomes of all occupational groups, with effect sizes indicating nontrivial differences, especially between NEET youth and post-secondary students (*d* = 0.40–0.50). This finding adds to a small number of longitudinal studies showing that youth NEET have compounded mental health risks even after accounting for initial differences (13).

However, the risks were not limited to youth NEET. Rather, among non-students, mental health only gradually improved as working hours increased, so that those working less than full-time had poorer mental health outcomes relative to peers in tertiary education, a finding that is not unique to the present study (34–36). Thus, finding a job is not sufficient to dramatically improve the mental health of young adults no longer in education— rather, working hours matter, and potentially working conditions more broadly (10, 15). Yet, the mental health needs of young adults no longer in education, especially if they fall outside the NEET category, have attracted much less attention than those of college and university students, who are more readily accessible to the research community (35, 37–39). As a result, students are often presented as a priority group for youth mental health interventions, even though non-students have generally worse mental health outcomes and less access to mental health services (5). For young adults not in education, employment can be a powerful drive of mental health for better or worse, depending on whether work is decent, meaningful, and supportive of identity development, versus insecure, casual, and insufficiently paid (2).

### Students in tertiary education have better mental health outcomes than their same-age peers in other occupations unless they work long hours

Students in tertiary education had better mental health outcomes on average than peers in vocational education or no longer in education, unless they worked long hours (> 20 h). For them, mental health worsened as working hours increased, so that not working or working limited hours seems to support mental health outcomes, in contrast to their peers no longer in education for whom low work hours seem to reflect precarity. Since the working hours-mental health associations held after accounting for initial differences, the findings add credence to previous cross-sectional observations suggesting a detrimental impact of working intensively on tertiary students’ mental health (24, 40). They also echo trends reported in a larger corpus focusing on educational outcomes, showing lower program completion rates among students in tertiary education working intensive hours vs. not (21). Together, these findings indicate that working longer hours is at least a marker of risk for both educational and mental health outcomes among students in tertiary education. As such, campus services should systematically consider financial needs and employment activities, and support healthy work-study balance among students. According to the results and to the work-enrichment scholarship more broadly, these services should strive to help students find study-related jobs likely to foster work-study enrichment rather than work-study conflicts (27).

### Students in vocational education, trade schools, or apprenticeship programs resemble workers more than tertiary students when it comes to associative patterns linking working hours and mental health

The mental health profile of young adults enrolled in vocational education, trade schools, or apprenticeship programs fell in between the other two groups, but tended to be closer to that of non-students than of students in tertiary education. For students in vocational programs, working hours were associated with favorable mental health outcomes like among non-students, although more weakly. These findings mirror those reported in a recent meta-analysis focusing on educational outcomes, which found that, for students in vocational institutions, working hours were associated with program completion in the German context (21).

## Implications for policy and practice

The results highlight one lever for promoting young adults’ mental health: supporting employment, at the right intensity, into quality jobs aligned with educational pursuits (15, 41). Such efforts are warranted for both those in education and those who have completed their studies. Among the latter, multicomponent interventions designed to improve youth NEET’s employment prospects have been found effective in this regard, although it is unclear whether they also improve mental health (42). Fewer intervention studies have specifically focused on underemployed or lesser employed youth, a group in dire need of more research and policy attention (16). For youth in tertiary education, several avenues could reduce employment encroachments on mental health. Findings from this study suggest that improving access to meaningful, study-related jobs might help somewhat, but not enough to offset the costs of intensive working hours. To that end, financial aid programs eliminating the need to work longer hours are likely necessary. However, attention to program design is warranted, as approaches significantly increasing student debt could induce financial stress and negatively affect student mental health (43). Also, few evidence-based programs exist to support the mental health of young adults in vocational, trades, and apprenticeship programs, but educational programming supporting the development of personal and social skills besides technical ones has shown promise (44). Finally, these findings highlight the relevance of developing strategies for improving study-life balance to support positive career development and mental health among young adults.

## Strengths and limitations

This study examined an understudied determinant of youth mental health in a representative sample, and comprehensively across students and non-students. Its longitudinal design is a strength in a field dominated by cross-sectional studies. Yet, its design remains correlational, thus precluding causal interpretations. Moreover, in the LISA data set, mental health information was limited to self-reported assessments. Also, information on job quality was limited in both depth and breadth. Notably, informal or unpaid work was not considered. Also, only a subsample (representing about 20% of the total) was questioned about their current job’s relatedness to their field of study, which limited capacity to flesh out its relevance. Furthermore, because stressors across life domains (work, education, life) were not assessed, underlying processes were not examined. Finally, the study focused on one social determinant of youth mental health: employment. However important, this represents only one among many determinants contributing to the youth mental health crisis. Future studies are needed to delineate how this aspect intersects with other key social determinants of health underlying inequities in employment.

## Conclusion

Working hours were associated with young adults’ mental health, although in contrasting ways depending on their position on the school-to-work transition continuum. The occupational groups with the most pressing mental health needs were unemployed and marginally employed youth no longer in education. As such, facilitating swift entry upon exiting education into stable employment in a decent, meaningful job should be part of any plan to alleviate the youth mental health crisis. Among youth in tertiary education, those working longer hours were also more at risk than their peers not working or working moderately, especially if their job was unrelated to their studies, showing that different types of jobs impact mental health differently. Improving tertiary students’ access to meaningful employment in study-enriching —rather than study-encroaching— job with a schedule calibrated to their needs should be a priority. Overall, the results reinforce previous calls for routinely integrating employment support into mental health services in Canada and highlight the need for integrated policies addressing youth employment and educational needs synergistically (9, 45).

## Data Availability

The data analyzed in this study is subject to the following licenses/restrictions: data and material from the Longitudinal International Study of Adults are only available from Statistics Canada for researchers who meet the criteria. Requests to access these datasets should be directed to www.statcan.gc.ca/en/microdata.
