# Supplementary figures and images for: Employment and the youth mental health crisis in Canada: distinct influences across phases of the school-to-work transition
Source: Front Public Health. 2025 Jul 31;13:1601463. doi: 10.3389/fpubh.2025.1601463 (PMC12350391; doi:10.3389/fpubh.2025.1601463)

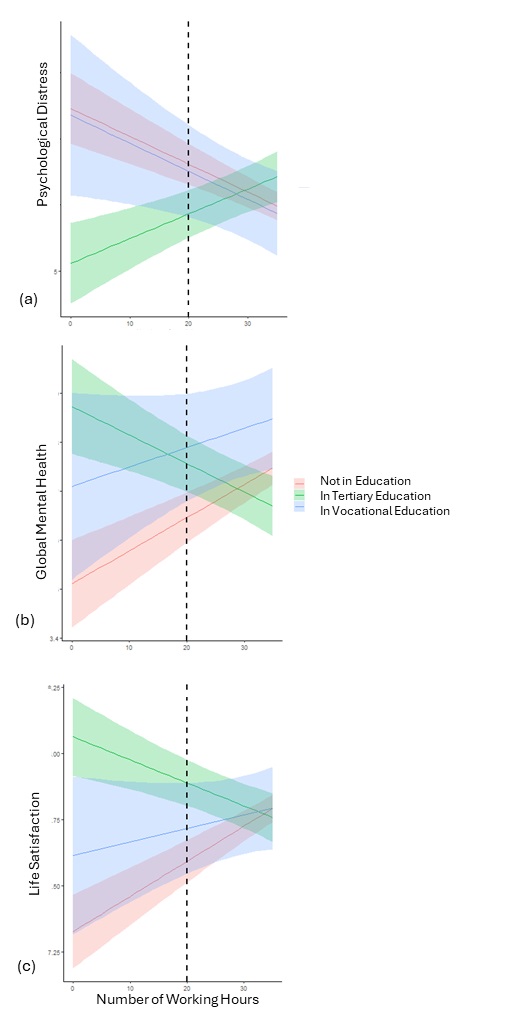

Supplement: Supplementary file 1 [file Image_1.jpeg]
